# X-ray computed tomography library of shark anatomy and lower jaw surface models

**DOI:** 10.1038/sdata.2017.47

**Published:** 2017-04-11

**Authors:** Pepijn Kamminga, Paul W. De Bruin, Jacob Geleijns, Martin D. Brazeau

**Affiliations:** 1 Naturalis Biodiversity Center Leiden, Darwinweg 2, Leiden 2333 CR, The Netherlands; 2 Institute Biology Leiden, Leiden University, Sylviusweg 72, Leiden 2333 BE, The Netherlands; 3 Department of Radiology, Leiden University Medical Center, Albinusdreef 2, Leiden 2300 RC, The Netherlands; 4 Department of Life Sciences, Imperial College London, Silwood Park Campus, Buckhurst Rd., Ascot SL5 7PY, UK

**Keywords:** Ichthyology, X-ray tomography, 3-D reconstruction

## Abstract

The cranial diversity of sharks reflects disparate biomechanical adaptations to feeding. In order to be able to investigate and better understand the ecomorphology of extant shark feeding systems, we created a x-ray computed tomography (CT) library of shark cranial anatomy with three-dimensional (3D) lower jaw reconstructions. This is used to examine and quantify lower jaw disparity in extant shark species in a separate study. The library is divided in a dataset comprised of medical CT scans of 122 sharks (Selachimorpha, Chondrichthyes) representing 73 extant species, including digitized morphology of entire shark specimens. This CT dataset and additional data provided by other researchers was used to reconstruct a second dataset containing 3D models of the left lower jaw for 153 individuals representing 94 extant shark species. These datasets form an extensive anatomical record of shark skeletal anatomy, necessary for comparative morphological, biomechanical, ecological and phylogenetic studies.

## Background & Summary

Computed tomography (CT) scanning has opened new ways for studying various parts of an organism’s biology. This technique has been used in sharks to elucidate the development^1^, function^2–5^ and morphology of their feeding mechanics^6–8^. The majority of these studies have focused on small taxonomic subsets to gain detailed anatomical knowledge. However, phylogenetically broad statistical analyses of shark cranial mechanics are still lacking. The lead author of this work is currently undertaking such studies. This contribution provides a descriptor of a large CT dataset of shark anatomy. We used whole specimens from museum collections (a few specimens only comprise the head due to their conservation) to create a dataset of medical CT scans which covers approximately 75% of all extant shark families^9^.

The x-ray computed tomography library presented here was created for investigating the ecomorphological diversity of shark feeding systems. The data from the CT scans were used to create a second dataset comprising three-dimensional (3D) models of the lower jaw. These models were used to examine lower jaw disparity in extant sharks species in a separate study by quantifying jaw shape using landmark based geometric morphometrics. The lower jaw was selected because it displays a diversity of jaw morphologies between shark species^10–15^. Lower jaws present biomechanically important features related to jaw closing mechanics and are therefore expected to function as a predictor of ecological specialisation^16–21^.

This tomography and virtual 3D dataset can be applied to or used to supplement further comparative and functional analyses of shark morphology. These may include investigations of (a)symmetry, development, integration and modularity or shape change through evolutionary time^22^. Furthermore, 3D anatomical models provide an excellent visual resource for outreach and education. For example, 3D models can be integrated into 3D PDF documents as interactive figures or can be physically reproduced using rapid prototyping (also referred to as stereolithography or 3D printing)^23^.

## Methods

### Specimens

122 individuals representing 73 extant species of 25 out of 34 families from all 9 orders of extant sharks^9^ (Selachimorpha, Chondrichthyes) are used in the CT scan dataset (Data Citation 1). The specimens are, at the time of CT scanning, formalin preserved and stored in 70% alcohol except for 4 specimens which were stored frozen (RMNH.PISC.36345, RMNH.PISC.verznr.2, 3 and 7). All CT scanned specimens are curated in the spirit collections of the British Museum of Natural History (BMNH) and Naturalis Biodiversity Center (NBC) (see the accompanying metadata for a complete list of specimens). The NBC collections comprise material from the Rijksmuseum van Natuurlijke Historie (RMNH) and the Zoölogisch Museum Amsterdam (ZMA); those institutional identifiers continue to be used here.

### CT scanning

CT scans of the specimens housed in the NBC collections were made at the Leiden University Medical Center, the Netherlands (LUMC) with a Toshiba Aquilion 64 medical scanner (Toshiba Medical Systems, Otawara, Japan) using a for the sharks customized scanning and reconstruction protocol (100 kV tube voltage, 150 mAs tube charge per rotation, 64 active channels, acquisition and reconstructed slice thickness 0.5 mm, reconstructed slice increment 0.5 mm, FC03 reconstruction filter, pitch factor 0.83). Specimens from the BMNH collection were scanned at the CT scanning facility of the Royal Brompton and Harefield NHS Trust (RBH), London, United Kingdom, with a Siemens Somatom Sensation 64 medical scanner using a customized scanning protocol with a variable reconstructed slice increment (100 kV tube voltage, 210 mAs tube charge per rotation, acquisition and reconstructed slice thickness 1.0 mm, B30f reconstruction filter).

### 3D segmentations

**Figure 1:**
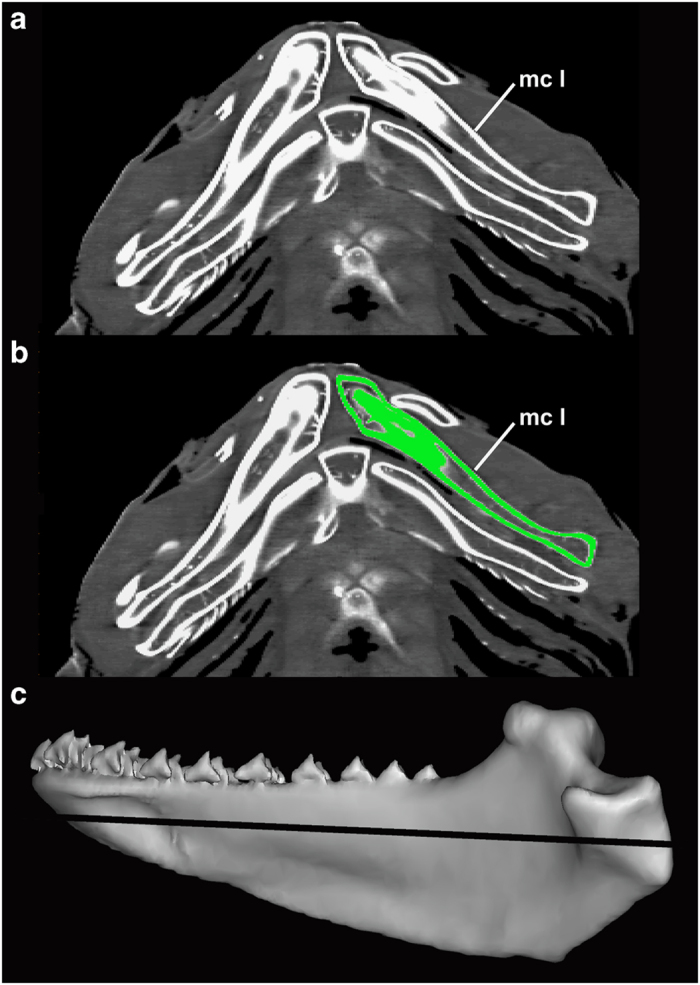
3D segmentation workflow in RMNH.PISC.24047 (*Squatina squatina*). (**a**) Each CT scan is build up of multiple slices (tomograms) showing structures with high attenuation of X-rays bright and structures with low attenuation dark, using a grey scale. Each pixel in the tomogram is associated with a Hounsfield unit. (**b**) A mask (green) is superimposed on the left lower jaw. This mask is based on predetermined threshold values of the Hounsfield unit of to produce an exact overlay on each tomogram. (**c**) When in each slice a mask is superimposed on the left lower jaw a 3D model is calculated based on the mask. The black line through the 3D model indicates the location of the tomogram in (**a**,**b**), mc l=left lower jaw.

The image series from the CT scans were imported into Mimics, v 15.01, (Materialise Software) for segmentation and 3D modelling. We used manual segmentation with a threshold edit of Hounsfield units (i.e., grey values) that are associated to the calcified cartilage of the lower jaw (Fig. 1). The Hounsfield units produced by the medical CT scanners are calibrated according to standard procedures of the manufacturer. The structures of calcified cartilage are not very dense in the scans (meaning relatively low hounsfield units, ranging from 400 to 1,500), and at the border between calcified cartilage and soft tissue, there will always be a partial volume effect that give a 'smoothed' transition from calcified cartilage to soft tissue. Structures with high density, such as the lower jaws, are depicted bright, while structures with low or intermediate density are depicted as dark using optimized greyscales (Fig. 1a). A mask based on measured threshold values of the Hounsfield units, which aligned very closely with the high relative density of the calcified cartilages of the lower jaw is set to produce an exact overlay on the slice images (Fig. 1b). We used these masks to create 3D models of the left lower jaws (Fig. 1c). Threshold values couldn’t be standardised across different scans due to variable tissue densities across specimens. Most 3D models could be modeled without ambiguity. Where manual thresholds were required, ambiguity was checked against left-right symmetry of the skeleton.

Subsequently, each 3D model of the left lower jaw was exported as a *.PLY file from MIMICS (Data Citation 1).

Thirty-one additional 3D models of the left lower jaw were segmented using CT scans provided by other researchers (Table 1), resulting in a total of 153 3D models of 94 extant shark species.

## Data Records

Data record 1—The data for this manuscript have been deposited in a Figshare repository (Data Citation 1). It comprises a dataset of 121 of volumes that consist of tomography images (‘slices’) in DICOM format reconstructed from medical computed tomography (CT) scans of sharks. The matrices of voxels in the dataset represent the Hounsfield units of the corresponding materials and tissues. The Hounsfield unit is associated with a well-defined physics quantity, being the linear attenuation coefficient of these materials and tissues. The majority of the data are whole body CT scans, while a few are CT scans of the head. From the CT scans we generated 153 3D models of the left lower jaws. The accompanying metadata describes the list of CT scanned specimens, their scan parameters and the derived 3D models included in Data record 1. Table 1 describes the specimen list and scan parameters of the additional 3D models of the left lower jaws that are created from CT scans provided by other researchers.

## Technical Validation

### CT scanner

Medical CT scanners are subject to a regular program for quality control and maintenance under the responsibility of a qualified medical physicist. However, discrepancies between the reconstructed values in an image and the true attenuation coefficients of the scanned object (i.e., image artifacts) can occur. Three common categories of CT image artefact appearances can be distinguished: streaking, shading, and rings and bands. Streaking artifacts appear as straight lines (bright and/or dark) across the image and are the result of the nature of the filtered backprojection reconstruction process. Shading artifacts can occur near objects of high contrast and usually appear in the soft tissue region near bony structures or near air pockets. This type of artifact is hard to identify since it shows a similar shape as the structure creating the shading. Ring and band artifacts can be visible as rings or bands overlaying the original image structure. The occurrence of artifacts can originate from the system design, x-ray tubes, detector, the specimen or operator^24^. In our dataset only two CT scans show significant artifacts. BMNH 1978.6.22.1 *Cetorhinus maximus* show ring artifacts in the dense vertebrae and BMNH 1978.6.22.1 *Cetorhinus maximus* shows ring and streaking artifacts in the dense vertebrae and posterior region of the head. Despite these artifacts skeletal structures were identifiable and useable for 3D segmentation. All other scans are free of artifacts or show only negligible artifacts.

The X-rays, generated by the CT scanner, are used to measure the transmission of X-ray through the specimens under hundreds of different angles. All these measurements are referred to as the raw data. This data is processed with a filtered backprojection, which generates a series of cross-sectional images^25^. Internal structures are visualized by their ability to attenuate the X-ray beams based on the linear attenuation coefficient. The parameters of the CT scanner were set to optimally visualize the jaws of the sharks. The jaws are well calcified compared to other skeletal structures in the head, therefore structures such as the neurocranium, basihyal and branchial chamber appear less clear in the scans.

## Usage Notes

The CT scan data in DICOM format can be loaded into 3D analysis software such as the free software package SPIERS^26^ or in license based software packages such as MIMICS (http://biomedical.materialise.com/mimics), AVIZO (http://www.fei.com/software/avizo3d/), or VG StudioMax (http://www.volumegraphics.com/en/products/vgstudio-max/basic-functionality/). 3D models of the structures of interest can be produced and exported in various formats using one of these software packages. The exported models can be used in landmark-based geometric morphometric methods^27,28^ to quantitatively test hypotheses of morphological diversity. The IDAV Landmark Editor^29^ is a free software package well suited for placing landmarks on the 3D models and exporting the landmark coordinates. Note that Landmark Editor only accepts *.PLY files without binary encoding. The software package Meshab (http://meshlab.sourceforge.net/) can be used to analyse, view and convert the *.PLY files to a usable format for Landmark Editor.

The landmark coordinates generated in Landmark Editor can be used in statistical software designed for geometric morphometric approaches, such as MorphoJ^30^, Morphologika^31^, PAST^32^ or the geomorph package^33^ in R^34^.

## Additional Information

**How to cite this article:** Kamminga, P. *et al.* X-ray computed tomography library of shark anatomy and lower jaw surface models. *Sci. Data* 4:170047 doi: 10.1038/sdata.2017.47 (2017).

**Publisher’s note:** Springer Nature remains neutral with regard to jurisdictional claims in published maps and institutional affiliations.

## Supplementary Material



## Figures and Tables

**Table 1:** Additional MC L.

**Registration number**	**Species**	**Family**	**Sex**	**TL (cm)**	**Scanned by**	**Institute**	**Scanner**	**Scan dose (kV)**	**Scan exposure time (mAs)**	**Algorithm**	**Slice increment (mm)**	**Slice thickness (mm)**
ERB 0854	Lamna ditropis	Lamnidae	f	234	F. Mollen	ZNA hospital Antwerp	Philips/Brilliance 40	120		B	0.5	1
ERB 0929	Lamna nasus	Lamnidae	m	174	F. Mollen	ZNA hospital Antwerp	Philips/Brilliance 40	120		B	0.5	1
ERB 0930	Lamna nasus	Lamnidae	m	166	F. Mollen	ZNA hospital Antwerp	Philips/Brilliance 40	120		B	0.5	1
ERB 0932	Carcharodon carcharias	Lamnidae	f	212	F. Mollen	ZNA hospital Antwerp	Philips/Brilliance 40	120		B	0.5	1
ERB 0933	Isurus oxyrinchus	Lamnidae	f	194	F. Mollen	ZNA hospital Antwerp	Philips/Brilliance 40	120		B	0.5	1
ERB 0934	Isurus oxyrinchus	Lamnidae	NA	230	F. Mollen	ZNA hospital Antwerp	Philips/Brilliance 40	120		B	0.5	1
ERB 0935	Isurus paucus	Lamnidae	f	254	F. Mollen	ZNA hospital Antwerp	Philips/Brilliance 40	120		B	0.5	1
ERB 0937	Lamna ditropis	Lamnidae	f	90	F. Mollen	ZNA hospital Antwerp	Philips/Brilliance 40	120		B	0.5	1
FLMNH 44978	Centroscymnus coelolepis	Somniosidae	f	NA	D. Huber	USF Center for Advanced Health	GE_Medical Systems/Lightspeed V	120	100	LUNG	0.625	0.625
LACMNH 3211	Cephaloscyllium ventriosum	Scyliorhinidae	NA	NA	M. Dean	UC Irvine Medical Center	Siemens/Sensation 16	120	196	H40f	0.699	0.75
LACMNH 38139	Stegostoma fasciatum	Stegostomatidae	NA	NA	M. Dean	UC Irvine Medical Center	Siemens/Sensation 16	120	196	H40f	0.699	0.75
LACMNH 38291	Chiloscyllium griseum	Hemiscylliidae	NA	NA	M. Dean	UC Irvine Medical Center	Siemens/Sensation 16	120	196	H40f	0.699	0.75
LACMNH 43856	Galeocerdo cuvier	Carcharhinidae	NA	NA	M. Dean	UC Irvine Medical Center	Siemens/Sensation 16	120	196	H40f	0.699	0.75
LACMNH 45857-1	Pseudocarcharias kamoharai	Pseudocarchariidae	f	NA	M. Dean	Toshiba America Medical Systems	Toshiba/Aquilion	120	125	FC04	variable	2
LACMNH 47362-1	Mitsukurina owstoni	Mitsukurinidae	NA	NA	M. Dean	Toshiba America Medical Systems	Toshiba/Aquilion	120	125	FC04	1	2
no id	Carcharias taurus	Odontaspididae	NA	NA	D. Huber	Tampa General Hospital	Philips/Brilliance 64	120		UA	variable	0.9
no id	Carcharhinus acronotus	Carcharhinidae	NA	NA	K. Mara; P. Motta, download from Digimorph	University Diagnostic Institute	Toshiba/Aquilion	120	100	FC30	0.5	1
no id	Eusphyra blochii	Sphyrnidae	NA	NA	K. Mara; P. Motta, download from Digimorph	University Diagnostic Institute	Toshiba/Aquilion	120	20	FC13	2	2
no id	Sphyrna mokarran	Sphyrnidae	NA	NA	K. Mara; P. Motta, download from Digimorph	University Diagnostic Institute	Toshiba/Aquilion	120	20	FC13	variable	1
no id	Sphyrna tudes	Sphyrnidae	NA	NA	K. Mara; P. Motta, download from Digimorph	University Diagnostic Institute	Toshiba/Aquilion	120	75	FC30	0.5	0.5
no id	Rhizoprionodon terraenovae	Carcharhinidae	NA	NA	K. Mara; P. Motta, download from Digimorph	University Diagnostic Institute	Toshiba/Aquilion 64	120	75	FC30	0.5	1
no id	Hexanchus griseus	Hexanchidae	NA	NA	D. Huber	USF Center for Advanced Health	GE_Medical Systems/Lightspeed V	120	100	LUNG	0.625	0.625
SIO CCS-79-4-5	Negaprion acutidens	Carcharhinidae	NA	NA	M. Dean	UC Irvine Medical Center					0.499	
SIO CCS-79-4-6	Negaprion acutidens	Carcharhinidae	NA	NA	M. Dean	UC Irvine Medical Center					0.5	
UF 160188	Alopias superciliosus	Alopiidae	NA	NA	R. Robins (provided by C. Crawford)	Medical University of South Carolina	Siemens/Sensation 64	80	287	B10s	0.6	0.6
USNM 3999	Orectolobus maculatus	Orectolobidae	f	NA	C. Crawford	Medical University of South Carolina	Siemens/Sensation 64	80	48	B10s	0.6	0.6
SIO 50-200	Carcharhinus brachyurus	Carcharhinidae	NA	NA	D. Walpole	Marine Vertebrate Collection, SIO	3T GE Signa Exite HDx				variable	0.7
SIO 62-1	Carcharhinus galapagensis	Carcharhinidae	NA	NA	C. Perry	Marine Vertebrate Collection, SIO	3T GE Signa Exite HDx				1	1
no id	Mustelus henlei	Triakidae	f	NA	R. Berquist	California Institute of Technology	7T Bruker Biospec Avance II				0.2	0.2
SIO 91-85	Orectolobus ornatus	Orectolobidae	NA	NA	A. Frew	Marine Vertebrate Collection, SIO	7T Bruker—UCLA				0.1	0.1
PSRC Uncat	Proscyllium habereri	Proscylliidae	NA	NA	R. Berquist	Pacific Shark Research Center	7T Bruker Biospec Avance II				0.1	0.1
